# Outcomes of hospitalized patients with COVID-19 during the course of the pandemic in a fully integrated health system

**DOI:** 10.1371/journal.pone.0263417

**Published:** 2022-02-25

**Authors:** Brian Lam, Maria Stepanova, Chapy Venkatesan, Ivan Garcia, Mary Reyes, Ashiq Mannan, Soleyah Groves, Mehul Desai, Andrei Racila, Andrej Kolacevski, Linda Henry, Lynn H. Gerber, Zobair M. Younossi

**Affiliations:** 1 Betty and Guy Beatty Center for Integrated Research, Inova Health System, Falls Church, VA, United States of America; 2 Inova Office of Research, Inova Health System, Falls Church, VA, United States of America; 3 Department of Medicine, Inova Health System, Falls Church, VA, United States of America; Stony Brook University Renaissance School of Medicine, UNITED STATES

## Abstract

**Background:**

Given the rapid spread of COVID-19 and its associated morbidity and mortality, healthcare providers throughout the world have been forced to constantly update and change their care delivery models.

**Objective:**

To assess the outcomes of COVID-19 hospitalized patients during the course of the pandemic in a well-integrated health system.

**Methods:**

The study used data from the electronic health medical records to assess trends in clinical profile and outcomes of hospitalized adult COVID-19 patients hospitalized in our 5-hospital health system from March 2020-May 2021 (n = 6865). Integration of the health system began in February 2020 and was fully actualized by March 30, 2020.

**Results:**

Mortality decreased from 15% during first peak (March-May 2020; the rate includes 19% in March-April and 10% in May 2020) to 6% in summer-fall 2020, increased to 13% during the second peak (November 2020-January 2021), and dropped to 7% during the decline period (February-May 2021) (p<0.01). Resource utilization followed a similar pattern including a decrease in ICU use from 35% (first peak) to 16% (decline period), mechanical ventilation from 16% (first peak, including 45% in March 2020) to 9–11% in subsequent periods (p<0.01). Independent predictors of inpatient mortality across multiple study periods included older age, male sex, higher multi-morbidity scores, morbid obesity, and indicators of severe illness on admission such as oxygen saturation ≤90% and high qSOFA score (all p<0.05). However, admission during the first peak remained independently associated with increased mortality even after adjustment for patient-related factors: odds ratio = 1.8 (1.4–2.4) (p<0.0001).

**Conclusions:**

The creation of a fully integrated health system allowed us to dynamically respond to the everchanging COVID-19 landscape. In this context, despite the increasing patient acuity, our mortality and resource utilization rates have improved during the pandemic.

## Introduction

The pandemic of COVID-19 reached the United States in early 2020 and soon created a tremendous burden for health systems [1, 2]. This was especially challenging for multi-hospital systems which were not fully integrated to muster intentional efforts as a unified healthcare organization to deal with COVID-19. For our multi-hospital health system, the COVID-19 pandemic compelled us to implement an integrated system-wide model of care delivery to deliver efficient and effective care against this deadly virus [3].

As the pandemic was unfolding outside the U.S., our health system had begun a system-wide initiation to transform its care delivery model from a holding company of individualistic clinical and business units within five separate hospitals to an integrated health system where the clinical enterprise became the centerpiece with eleven comprehensive, program-based clinical service lines [3]. Early in the pandemic, we mobilized and responded within the context of this new model of care so that the eleven clinical service lines worked closely with our five hospitals to act as a unified and integrated health system for managing patients hospitalized with COVID-19 infection. This “system-ness” approach combined the knowledge, capabilities, learning, and resources within system created our playbook for managing COVID-19 [3].

Given the nature of COVID-19 as a disease, the Medicine Service line which included system divisions for Hospitalist Medicine, Critical Care Medicine, Emergency Medicine, and Infectious Disease Medicine, was primarily responsible for the care of these patients. In this context, our health system quickly responded by creating a staffing model, dedicated COVID-19 units, treatment algorithms, a central communication hub, and organizing the provision of other resources needed to meet this unprecedent challenge [3].

Over the first 18 months of COVID-19 experience, the pandemic in our region has followed a heterogeneous trend with distinct peaks in hospitalizations and deaths. We experienced the initial peak of COVID-19 in early 2020 (the first peak) which was followed by a relatively low infection rate in the summer and early fall of 2020 (the plateau), then by an increase in infections and hospitalizations in late fall and early winter of 2020–2021 (the second peak), and then a steady decline in both during spring of 2021 which occurred concurrently with the increasing vaccination rates in our region until the arrival of the more infectious B.1.617 (Delta) strain in the summer of 2021. Over the course of this pandemic, so far, in addition to implementing new models of care, both patient populations hospitalized with COVID-19 and treatments have been evolving, each likely making additional contributions to the outcomes [4, 5].

Our aim was to assess the outcomes of COVID-19 hospitalized patients during the first 15 months of the pandemic in a well-integrated health system.

## Methods

Data were collected from electronic medical records (EMRs) of adults (≥18 years) with a diagnosis of COVID-19 (ICD-10 code U07.1) who were hospitalized in our health system between March 5^th^, 2020, and June 1^st^, 2021 and had a discharge status at the time of the analysis. The health system includes five hospitals with a total of 1,800 licensed acute care beds, located in two of the largest counties of Virginia, USA. Given the limitations of EMRs, each case was also reviewed manually by trained personnel to ensure the reliability of the extraction process as well as to confirm data accuracy and completeness. Discussion of integrating our five individual hospitals into one integrated hospital system began in late 2019 with the initial steps starting in February 2020 just prior to the onset of the COVID-19 pandemic.

The definitions used in this study were as follows: Race/ethnicity was classified into non-Hispanic white (whites), non-Hispanic black (blacks), Hispanic, Asian, and other/biracial groups. Congregated living settings included skilled nursing facilities, residential and other long-term care facilities, or rehabilitation facilities. Charlson comorbidity index (CCI) and Elixhauser comorbidity index (ECI) scores both of which are widely used in clinical practice to predict comorbid patients’ risk of mortality were calculated for each patient using their medical history [6, 7]. In addition, vital signs data collected at admission were used to calculate the quick sequential organ failure assessment (qSOFA) score [8]. Obesity was defined as a body mass index (BMI) ≥ 30 kg/m2 while morbid obesity was defined as a BMI ≥ 40 kg/m2.

Study outcomes included inpatient mortality and resource utilization, such as length of hospital stay (in days), admission to the intensive care unit (ICU), the use of mechanical ventilation, and ECMO.

### Statistical analysis

Based on the trends in COVID-19-related hospital admissions in our health system, we defined the months of March–May 2020 as the first peak of inpatient hospitalizations and November 2020 –January 2021 as the second peak. The period of June–October 2020 was considered a plateau between the two peaks, and the period of February-May 2021 was considered a decline.

Patients’ parameters were summarized as N (%) or mean (±SD). Parameters were compared between groups using χ2 or Kruskal-Wallis tests for categorical or continuous parameters, respectively. For patients with multiple admissions, demographic and clinical parameters collected at the time of the first admission were used, along with the outcome of the last admission, and the total cumulative resource utilization across all admissions. Logistic regression models were used to identify clinical, demographic, and laboratory parameters associated with outcomes. P-values <0.05 were considered statistically significant.

SAS 9.4 (SAS Institute, Cary, NC) was used for all analyses. The study was granted a waiver of consent and an exemption status by the Inova Health System’s Institutional Review Board given that all data were deidentified and analyzed anonymously.

## Results

During the study period, 6865 patients who had a diagnosis of COVID-19 were discharged from our health system: age 58±19 years; 51% male; 27% white; 16% black; 42% Hispanic; 11% Asian; 9% from congregated living; 9% with morbid obesity; baseline CCI score, 3.8±3.5; baseline ECI, 10.4±11.4.

### Changes in the profile of hospitalized patients over time

**Fig 1:**
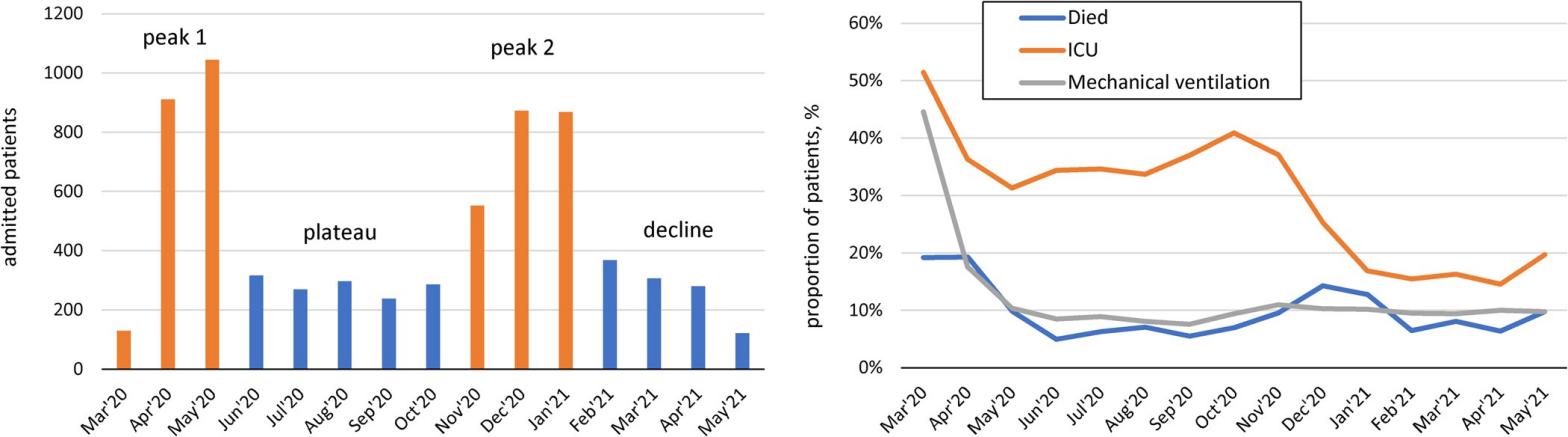
Monthly trends in the number of admissions, resource utilization, and mortality.

Of the study cohort, 2086 patients were admitted during the first peak (March–May, 2020), 1407 were admitted during the plateau (June–October 2020) while 2295 patients were admitted during the second peak (November 2020–January 2021), and 1077 were admitted during the decline period (February-May 2021) (**Fig 1**, **Table 1)**. From the first peak to the plateau period, the mean age of hospitalized patients decreased (from 59 years to 52 years), then increased again to mean 62 years during the second peak, and then decreased to 55 years during the decline period. The percentage of male patients also changed, from 55% to 45% to 52% to 50%, as did the racial distribution. The most substantial change was that as the proportions of non-Hispanic whites admitted (21% to 20% to 33% to 32%) increased the proportion of Hispanics admitted decreased (52% to 52% to 32% to 28%) inpatients (all p<0.01). The proportion of patients living in congregated settings was at least two-fold higher during the first peak in comparison to later two periods (17% vs. 7% vs. 5%) and decreased to zero during the decline period (p<0.0001) (**Table 1**). Patients tended to present with more severe hypoxia during peak periods based on their oxygen saturation measured at admission (**Table 2**).

**Table 1:** Clinical and demographic characteristics of patients admitted to hospitals of Inova Health System by the period of admission.

	Peak 1 (March-May 2020)	Plateau (June-October 2020)	Peak 2 (November 2020-January 2021)	Decline (February-May 2021)	p	All
N	2086	1407	2295	1077		6865
Inova Alexandria hospital	426 (20.4%)	256 (18.2%)	420 (18.3%)	224 (20.8%)	0.12	1326 (19.3%)
Inova Fair Oaks hospital	198 (9.5%)	105 (7.5%)	246 (10.7%)	107 (9.9%)	0.0122	656 (9.6%)
Inova Fairfax hospital	1059 (50.8%)	713 (50.7%)	1009 (44.0%)	460 (42.7%)	< .0001	3241 (47.2%)
Inova Loudon hospital	226 (10.8%)	220 (15.6%)	390 (17.0%)	192 (17.8%)	< .0001	1028 (15.0%)
Inova Mount Vernon Hospital	177 (8.5%)	113 (8.0%)	230 (10.0%)	94 (8.7%)	0.15	614 (8.9%)
Age, years (mean ± SD)	58.9 ± 18.2	52.3 ± 19.3	61.6 ± 18.9	55.2 ± 19.1	< .0001	57.9 ± 19.1
Age < 45	486 (23.3%)	528 (37.5%)	458 (20.0%)	334 (31.0%)	< .0001	1806 (26.3%)
Age 45–54	389 (18.6%)	246 (17.5%)	347 (15.1%)	186 (17.3%)	0.0182	1168 (17.0%)
Age 55–64	417 (20.0%)	223 (15.8%)	411 (17.9%)	199 (18.5%)	0.0195	1250 (18.2%)
Age 65–74	338 (16.2%)	196 (13.9%)	419 (18.3%)	171 (15.9%)	0.0064	1124 (16.4%)
Age 75–84	268 (12.8%)	147 (10.4%)	397 (17.3%)	109 (10.1%)	< .0001	921 (13.4%)
Age 85–94	147 (7.0%)	61 (4.3%)	228 (9.9%)	65 (6.0%)	< .0001	501 (7.3%)
Age ≥ 95	41 (2.0%)	6 (0.4%)	35 (1.5%)	13 (1.2%)	0.0017	95 (1.4%)
Male	1152 (55.2%)	637 (45.3%)	1197 (52.2%)	536 (49.8%)	< .0001	3522 (51.3%)
Non-Hispanic White or Caucasian	437 (21.4%)	269 (19.5%)	739 (32.8%)	318 (32.1%)	< .0001	1763 (26.5%)
Non-Hispanic black or African-American	298 (14.6%)	215 (15.6%)	323 (14.4%)	212 (21.4%)	< .0001	1048 (15.7%)
Hispanic	1054 (51.7%)	721 (52.2%)	703 (31.8%)	293 (28.1%)	< .0001	2771 (41.5%)
Asian	181 (8.9%)	90 (6.5%)	311 (13.8%)	129 (13.0%)	< .0001	711 (10.7%)
Other race/ethnicity	87 (4.3%)	97 (7.0%)	187 (8.3%)	83 (8.4%)	< .0001	454 (6.8%)
Congregated living	361 (17.3%)	92 (6.5%)	119 (5.2%)	0 (0.0%)	< .0001	572 (8.6%)
BMI, kg/m2	29.6 ± 7.2	30.7 ± 7.1	29.7 ± 7.6	30.4 ± 7.8	< .0001	30.0 ± 7.4
BMI <18	38 (1.9%)	15 (1.1%)	42 (1.9%)	25 (2.4%)	0.12	120 (1.8%)
Obesity (BMI ≥ 30)	804 (40.8%)	644 (48.3%)	913 (41.7%)	459 (44.3%)	0.0001	2820 (43.2%)
Morbid obesity (BMI ≥ 40)	163 (8.3%)	117 (8.8%)	190 (8.7%)	93 (9.0%)	0.91	563 (8.6%)
Charlson comorbidity index (CCI)	3.93 ± 3.59	3.02 ± 3.35	4.24 ± 3.52	3.43 ± 3.55	< .0001	3.77 ± 3.54
CCI = 0	366 (17.5%)	432 (30.7%)	324 (14.1%)	258 (24.0%)	< .0001	1380 (20.1%)
CCI = 1	317 (15.2%)	190 (13.5%)	285 (12.4%)	172 (16.0%)	0.0119	964 (14.0%)
CCI = 2	264 (12.7%)	180 (12.8%)	282 (12.3%)	137 (12.7%)	0.97	863 (12.6%)
CCI = 3 or 4	376 (18.0%)	233 (16.6%)	474 (20.7%)	186 (17.3%)	0.0075	1269 (18.5%)
CCI = 5–8	497 (23.8%)	244 (17.3%)	622 (27.1%)	203 (18.8%)	< .0001	1566 (22.8%)
CCI ≥ 9	266 (12.8%)	128 (9.1%)	308 (13.4%)	121 (11.2%)	0.0006	823 (12.0%)
Elixhauser comorbidity index (ECI)	11.2 ± 11.5	8.28 ± 11.02	11.3 ± 11.2	9.49 ± 11.59	< .0001	10.4 ± 11.4
ECI ≤ 0	448 (21.5%)	460 (32.7%)	462 (20.1%)	297 (27.6%)	< .0001	1667 (24.3%)
1 ≤ ECI ≤ 5	459 (22.0%)	338 (24.0%)	483 (21.0%)	252 (23.4%)	0.15	1532 (22.3%)
6 ≤ ECI ≤ 10	283 (13.6%)	173 (12.3%)	322 (14.0%)	133 (12.3%)	0.35	911 (13.3%)
11 ≤ ECI ≤ 17	357 (17.1%)	179 (12.7%)	421 (18.3%)	166 (15.4%)	0.0001	1123 (16.4%)
18 ≤ ECI ≤ 27	313 (15.0%)	156 (11.1%)	378 (16.5%)	128 (11.9%)	< .0001	975 (14.2%)
ECI ≥ 28	226 (10.8%)	101 (7.2%)	229 (10.0%)	101 (9.4%)	0.0034	657 (9.6%)

**Table 2:** Vital signs, symptoms and laboratory parameters on admission.

	Peak 1 (March-May 2020)	Plateau (June-October 2020)	Peak 2 (November 2020-January 2021)	Decline (February-May 2021)	p	All
Blood pressure diastolic, mmHg	73.1 ± 13.3	72.1 ± 12.5	72.3 ± 12.8	72.9 ± 12.5	0.06	72.6 ± 12.9
Blood pressure systolic, mmHg	129.4 ± 23.7	126.7 ± 21.7	129.5 ± 23.6	128.7 ± 22.7	0.0004	128.8 ± 23.2
Temperature, degrees F	99.2 ± 1.7	98.8 ± 1.3	98.7 ± 1.4	98.6 ± 1.3	< .0001	98.9 ± 1.5
Heart rate per minute	92.8 ± 19.9	86.4 ± 18.7	87.0 ± 19.9	87.3 ± 18.8	< .0001	88.7 ± 19.7
Respiratory rate per minute	23.2 ± 8.1	21.3 ± 7.0	22.4 ± 7.4	22.0 ± 7.6	< .0001	22.4 ± 7.6
Oxygen saturation, %	92.4 ± 7.9	94.0 ± 6.7	92.6 ± 7.1	93.6 ± 6.9	< .0001	93.0 ± 7.3
Low oxygen saturation (≤ 90%)	489 (23.6%)	237 (17.2%)	598 (26.1%)	222 (20.7%)	< .0001	1546 (22.7%)
On supplemental oxygen at admission	1723 (83.2%)	1245 (90.2%)	1870 (81.6%)	919 (85.8%)	< .0001	5757 (84.5%)
High risk (qSOFA ≥ 2)	165 (7.9%)	71 (5.1%)	200 (8.7%)	51 (5.9%)	0.0001	487 (7.3%)
Laboratory parameters						
ALT, U/L	50.6 ± 77.8	50.7 ± 77.6	47.0 ± 55.3	49.8 ± 98.7	0.18	49.3 ± 74.6
AST, U/L	61.0 ± 108.3	53.2 ± 74.1	55.8 ± 69.3	58.3 ± 84.1	< .0001	57.3 ± 86.3
Bicarbonate, mEq	22.5 ± 3.8	22.6 ± 4.0	22.8 ± 4.0	22.8 ± 4.6	0.18	22.7 ± 4.0
Serum creatinine, mg/dL	1.51 ± 1.91	1.30 ± 1.53	1.44 ± 1.81	1.39 ± 1.58	< .0001	1.43 ± 1.76
C-reactive protein, mg/L	12.6 ± 9.3	10.8 ± 8.5	10.9 ± 8.2	10.6 ± 8.2	< .0001	11.4 ± 8.7
D-dimer, mg/L	2.30 ± 6.17	2.04 ± 3.46	2.16 ± 3.29	2.28 ± 3.97	0.0002	2.20 ± 4.58
Ferritin, ng/mL	1310.1 ± 1943.7	952.8 ± 1446.7	1159.6 ± 1768.5	1157.2 ± 1845.9	< .0001	1171.3 ± 1789.6
Hemoglobin, g/dL	13.2 ± 2.5	12.9 ± 2.0	13.0 ± 2.1	12.9 ± 2.2	0.0001	13.0 ± 2.2
Absolute lymphocyte count	1.31 ± 3.41	2.09 ± 10.58	1.19 ± 4.19	3.20 ± 21.74	< .0001	1.65 ± 8.67
Platelet, 109/L	232.3 ± 95.3	233.8 ± 92.5	228.5 ± 94.1	236.3 ± 98.3	0.07	231.5 ± 94.5
Total Bilirubin, mg/dL	0.652 ± 0.740	0.692 ± 1.048	0.701 ± 1.021	0.696 ± 0.980	0.0151	0.683 ± 0.941
White blood count, 109/L	8.84 ± 18.51	9.36 ± 21.70	8.15 ± 4.48	9.51 ± 17.55	< .0001	8.75 ± 15.86

### Changes in the outcomes of hospitalized COVID-19 patients

Mortality decreased from 15% during peak 1 (which included 19% in the months of March and April 2020 followed by the most rapid decline to 10% in May 2020) to 6% during the plateau period (varied between 5%–7% across months), and then increased to 13% in the second peak (**Table 3**, **Fig 1**). Assessment of monthly trends did not return statistically significant differences between the months within those periods after April 2020 (**Fig 1**). In contrast, the proportion of patients discharged to their homes increased from 71% during peak 1 (65–66% in March–April and 77% in May) to 85% during the plateau period, and then decreased to 73% during peak 2 followed by 80% during the decline period (**Table 3**).

**Table 3:** Resource utilization and outcomes of COVID-19 patients admitted to Inova Health System hospitals.

	Peak 1 (March-May 2020)	Plateau (June-October 2020)	Peak 2 (November 2020-January 2021)	Decline (February-May 2021)	p	All
Length of stay, days	10.5 ± 11.0	8.02 ± 9.76	9.15 ± 10.01	7.63 ± 8.77	< .0001	9.10 ± 10.14
Admitted to ICU	725 (34.8%)	507 (36.0%)	573 (25.0%)	172 (16.0%)	< .0001	1977 (28.8%)
Received mechanical ventilation	327 (15.7%)	120 (8.5%)	240 (10.5%)	104 (9.7%)	< .0001	791 (11.5%)
Received ECMO	29 (1.4%)	8 (0.6%)	15 (0.7%)	4 (0.4%)	0.0048	56 (0.8%)
Inpatient hospice care at any point	127 (6.1%)	17 (1.2%)	81 (3.5%)	23 (2.1%)	< .0001	248 (3.6%)
Readmission	105 (5.0%)	69 (4.9%)	110 (4.8%)	38 (3.5%)	0.26	322 (4.7%)
Discharged to:						
Short-term care facility	9 (0.4%)	12 (0.9%)	16 (0.7%)	23 (2.1%)	< .0001	60 (0.9%)
Long-term care facility	239 (11.5%)	96 (6.8%)	259 (11.3%)	99 (9.2%)	< .0001	693 (10.1%)
Home	1484 (71.1%)	1192 (84.7%)	1670 (72.8%)	857 (79.6%)	< .0001	5203 (75.8%)
Hospice care	50 (2.4%)	20 (1.4%)	61 (2.7%)	19 (1.8%)	0.06	150 (2.2%)
Died	304 (14.6%)	87 (6.2%)	289 (12.6%)	79 (7.3%)	< .0001	759 (11.1%)

There were heterogeneous trends in resource utilization over time. The most significant change was in the use of mechanical ventilation which decreased from 16% during peak 1 to 9–11% during subsequent periods. The rate of ICU utilization remained relatively stable at 35–36% during both peak 1 and plateau periods followed by 25% during peak 2 and 16% during the decline period (**Table 3**, **Fig 1**). The total length of inpatient stays also decreased from a mean of 10.5 days during peak 1 to 8–9 days in later periods (**Table 3**). Monthly trends suggested that while the use of mechanical ventilation was as high as 45% in March, it decreased to 18% in April, 10% in May, and then stabilized at the rate of roughly 10% (**Fig 1**). Other resource utilization parameters were also at their highest in the month of March and decreased over time albeit less steeply.

### Factors associated with death of COVID-19 inpatients

In multivariate logistic regression analysis, adjusted for patient-related factors, inpatient mortality was independently associated with hospital admission during the first peak period: odds ratio (OR) (95% CI) = 1.8 (1.4–2.4), p<0.0001, with reference to the plateau period. The same ORs for the second peak and for the decline period were not statistically significant (p>0.05) (**Table 4**). However, without adjustment for patient-related factors, the ORs for mortality in each time period were 2.6 (2.0–3.3) for the first peak; 2.2 (1.7–2.8) for the second peak; 1.2 (0.9–1.6) for the decline period. Other predictors of inpatient mortality in the multivariate model were older age, male sex, morbid obesity, higher baseline ECI scores, lower oxygen saturation and higher qSOFA score at admission (all p<0.01) (**Table 4**). On the other hand, inpatient mortality was not associated with race/ethnicity (all P>0.05) (**Table 4**). Although a higher ECI score was associated with mortality, no single comorbid condition, except for morbid obesity, was independently associated with mortality (all *P*>0.05).

**Table 4:** Independent predictors of inpatient mortality in patients with COVID-19 across the entire study period.

predictor	OR (95% CI)	p
Admission during peak 1 (ref: plateau 2020)	1.83 (1.38–2.44)	< .0001
Admission during peak 2 (ref: plateau 2020)	1.28 (0.96–1.70)	0.09
Admission during decline 2021 (ref: plateau 2020)	0.81 (0.55–1.20)	0.29
Age, per 5 years	1.21 (1.17–1.25)	< .0001
Male gender (ref: female)	1.37 (1.14–1.65)	0.0010
Hispanic (ref: white)	1.01 (0.79–1.29)	0.94
Black (ref: white)	0.87 (0.66–1.14)	0.32
Asian (ref: white)	1.00 (0.74–1.34)	0.98
Morbid obesity	2.15 (1.52–3.04)	< .0001
ECI 6–10 (ref: ECI ≤ 5)	3.25 (2.15–4.90)	< .0001
ECI 11–17 (ref: ECI ≤ 5)	6.91 (4.82–9.91)	< .0001
ECI 18–27 (ref: ECI ≤ 5)	10.19 (7.09–14.64)	< .0001
ECI ≥ 28 (ref: ECI ≤ 5)	17.07 (11.77–24.76)	< .0001
Oxygen saturation at admission ≤ 90%	2.73 (2.25–3.32)	< .0001
High risk (qSOFA≥2 at admission)	1.98 (1.54–2.54)	< .0001

When predictors of mortality during the first and second peak periods were studied separately (**S1 Table**), we found that factors independently associated with inpatient mortality remained similar (all p>0.05 for the interaction terms) with the only exception being morbid obesity: OR = 2.8 during peak 1 and OR = 1.2 during peak 2 (p = 0.022 for the respective interaction term). Indeed, while among patients without morbid obesity, the mortality rates were similar between the two peaks (14% vs. 13%, p = 0.28), the same rates for patients with morbid obesity were 17% during peak 1 vs. 9% during peak 2 (p = 0.046).

### Racial and ethnic differences among COVID-19 patients

In the study cohort, 27% of patients were non-Hispanic white, 16% were non-Hispanic black, 42% were Hispanic, 11% were Asian, and 7% were biracial/other. The proportion of Hispanic patients was the highest during the plateau period and the lowest during the decline period (28%) (**Table 1**). A CCI score of 0 was observed in 9% of white patients, 13% of black patients, 32% of Hispanic patients, and 12% of Asian patients (*P*<0.0001) (**S2 Table**).

Because more than 40% of admitted patients were Hispanic, we additionally compared them to other patients (**S3 Table**). Compared to non-Hispanic patients, Hispanic inpatients had a lower mean age (49 vs 64 years for non-Hispanic), 41% vs. 16% were younger than 45 years of age, were less likely to live in congregated setting (1.4% vs 14% for non-Hispanic), had fewer comorbidities (mean CCI 2.3 vs. 4.9, mean ECI 6.6 vs 13.1) (all *P*<0.01). Despite this, Hispanic patients presented with higher temperatures and heart rates, lower levels of oxygen saturation, higher mean CRP levels, and more commonly required treatment with supplemental oxygen at admission (all p<0.01). None the less, Hispanic patients had lower mortality (6.4%) compared to non-Hispanic patients (14.3%). However, after accounting for their younger age and lower baseline comorbidity index, multivariate analysis did not return any significant association between mortality and race/ethnicity (all *P*>0.05) (**Table 4**).

### Baseline multi-morbidity scores and inpatient mortality

Although patients who died had substantially more comorbidities than those who survived, no individual comorbidity (except for morbid obesity) was found to be independently associated with mortality (all *P*>0.05). However, CCI and ECI scores were each associated with inpatient mortality (**Table 4**). In fact, only 1.2% of patients with CCI scores of 0–1 died while mortality increased to 8.0% for patients with CCI scores of 3–4, 21% for patients with CCI scores of 5–8, and to 28% for patients with CCI scores ≥ 9. A similar trend was observed with increasing ECI scores (**S4 Table**).

## Discussion

We analyzed data from a large cohort of inpatients with COVID-19 from our health system over the entire course of the pandemic. In the context of this pandemic, we were compelled to accelerate our transformation into a fully integrated health system to rapidly meet the emergent needs of the patients and our health care providers. As we integrated our health system, our mortality and hospital resource utilization improved. In fact, mortality of inpatients with COVID-19 decreased from nearly 20% in early spring of 2020 to 6% in summer of 2020, then increased to 13% during the second peak, and then dropped again to 7% during the decline period.

Although a number of factors may have contributed to higher inpatient mortality earlier during the pandemic (older age and higher proportion of patients residing in the congregated living settings) [9–11], some factors were related to changes that were implemented by our health system. These changes included creation of systemwide critical care, hospital medicine and infectious disease services. In this context, we have tried to untangle the contributions of patient-related vs. management-related factors that may have affected patients’ outcomes.

Our data confirmed that older age, male sex, higher comorbidity scores, lower oxygen saturation and higher qSOFA scores were independently associated with inpatient mortality in the multivariate model [12–15]. Nevertheless, even after accounting for these factors, being admitted during the first peak period of the pandemic remained independently associated with higher mortality. In this context, it is important to note that no such association was found for admissions during the second peak of COVID-19 or during the decline period. These data suggest that after the initial peak of the pandemic, our integrated care became more robust so that drivers of subsequent reduction in mortality were largely limited to patient-related factors.

In addition to age and disease severity, we also assessed the role of ethnicity on the outcome. Consistent with national reports, we showed that hospitalized COVID-19 patients were disproportionately Hispanic, accounting for more than 40% of our inpatient sample which is significantly higher than their share of 16% in our region [16]. Although we did not find increased mortality rates once hospitalized for any ethnic group, our data confirm that Hispanic population bears a higher burden of being hospitalized for COVID-19 and, therefore, continues to need increased community outreach support about COVID-19, when to seek care, and encouragement to get vaccinated.

In this study, we also found that multimorbidity scores (both CCI and ECI) were consistently and strongly associated with inpatient mortality. In fact, the ORs for dying from COVID-19 steadily increased for patients with higher comorbidity scores. Although we found that morbid obesity was independently associated with mortality, other individual comorbidities were no longer associated with mortality after adjustment for age. This finding might shed some light on inconsistencies previously reported about mortality risk factors such that multimorbidity rather than individual comorbidities need to be considered to accurately predict COVID-related inpatient mortality [15–18].

Since the association of admission during the first few months of the pandemic with a higher risk of inpatient mortality remained statistically significantly even after adjustment for patient-related factors, we believe that changes in the clinical care of inpatients as a result of integration of our health system may have played an important role in the improvement of outcomes and decreasing hospital resource utilization. In this context, we carried out a number of major transformations in delivery of inpatient care for these patients across our healthcare system which is fully explained in an earlier publication [3]. Briefly, our system integration allowed for the standardization of care that was coordinated with a team approach. Our Critical Care COVID-19 Strike Team created a systemwide approach to all aspects of care for patients with COVID-19. This team also modified and made more restrictive the criteria for when COVID-19 patients were to be placed on mechanical ventilation. This change is reflected in the data showing the rate of mechanical ventilation in this study decreased from 45% in March 2020 to <10% in June 2020 which has remained stable since then.

The Medicine Hospitalists created multidisciplinary early recognition and intervention teams (MERIT) which are/were collaborative teams comprised of hospitalists, nurses, respiratory therapists, and subspecialty groups (critical care/pulmonology and infectious disease). From the MERIT formation, two different COVID 19 units were developed- a COVID-19 unit to deliver standard care to COVID-19 patients and a COVID-19 step down augmented unit to care for the sicker patients. Both of these floor units were scalable and defined, in part, by the maximum level of oxygen therapy required and available. In addition, a case review team consisting of a hospitalist, a charge nurse, and a floor pulmonologist assigned to a COVID-19 unit rounded three times daily to provide early identification and intervention for patients with COVID-19 who were at a heightened risk of deterioration in order to mitigate the need for ICU transfer and/or mechanical ventilation [3]. Combined these actions may better help to understand some of the phenomenon seen in our study. For example, ICU use was highest during the plateau phase as a result of the specialty COVID-19 units scaling back their capacity which then caused an increase in the ICU census. However, as the second peak began, the COVID-19 specialty units scaled back up their operations which put less demand on the ICU’s.

We have used our experience with COVID-19 pandemic and full integration of our health system to begin important discussions within our community regarding our response to potential future surges of this pandemic or other similar future healthcare crisis. In this context, there are certain relatively fixed risk factors such as patient characteristics that can be associated with adverse outcomes. On the other hand, we believe that potentially modifiable factors can be quickly instituted through an integrated care delivery model which can provide standardized treatment algorithms, efficiently adopt best critical care practices, create specialized care units, quickly expand the provider force, and acquire new drugs, laboratory tests, and personal protective equipment as needed. We believe that our integrated care delivered by our five hospitals though our service line model was responsible for the optimal care of our patients.

A limitation of the study is its observational cross-sectional design, so we cannot establish a cause-and-effect relationship between risk factors and outcomes. Another limitation is that post-discharge data were not available, so no conclusions about COVID-associated morbidity or mortality can be made for patients after they were discharged. Furthermore, no conclusions can be made about COVID-19 patients who were not admitted to a hospital; this includes patients who were treated with monoclonal antibodies in an outpatient setting, other patients who were discharged after visiting ER or were admitted for observation only, and also patients who lacked access to hospital care altogether. The clinical and laboratory data available for analysis from EMRs and chart review allowed testing only a limited number of hypotheses so future work is needed to come up with better strategies for inpatient treatment of COVID-19 which would further improve outcomes. The study does not cover most of the period when vaccines became widely available in our region and also subsequent surges of infections with emerging COVID-19 strains. The strength of this study is the large volume of patients admitted to an integrated health system and the amount of data systematically collected over 15 months of the pandemic covering both peaks of COVID-19 infections in 2020, so meaningful changes and trends could be identified.

In summary, our in-depth analysis of patients hospitalized with COVID-19 in our health system documented the benefits of implementing an integrated health system based on service line model of care to deliver efficient, seamless and rapid care to patients with COVID-19 throughout our system. Although some improvement of the outcomes were related to changes in patient profiles, additional improvements were observed because of the coordination of care through fully integrated care delivery. These findings may provide insights for dealing with challenges of large-scale needs during health care crisis that can be delivered in an organized and integrated model of healthcare.

## Supporting information

S1 Table Independent predictors of inpatient mortality in patients with COVID-19 across in different study periods separately. (DOCX)

S2 Table Comparison of clinico-demographic parameters and outcomes in patients of different races/ethnicities hospitalized with COVID-19. (DOCX)

S3 Table Comparison of clinico-demographic parameters and outcomes in Hispanic vs. non-Hispanic patients hospitalized with COVID-19. (DOCX)

S4 Table Comparison of outcomes of patients with different baseline comorbidity scores. (DOCX)

S1 File Deidentified HIPAA-compliant patient-level data used in this study. (XLSX)

## Abbreviations

SARS-CoV-2: severe acute respiratory syndrome coronavirus 2
EMR: electronic medical record
BMI: body mass index
CCI: Charlson’s comorbidity index
ECI: Elixhauser’s comorbidity index
qSOFA: quick Sequential Organ Failure Assessment
ICU: intensive care unit
ECMO: extracorporeal membrane oxygenation
OR: odds ratio
CRP: C-reactive protein
TIA: transient ischemic attack
CVA: cerebrovascular accident
ALT: alanine aminotransferase
AST: aspartate aminotransferase
