# Supplementary material for: Outcomes of hospitalized patients with COVID-19 during the course of the pandemic in a fully integrated health system
Source: PLoS One. 2022 Feb 25;17(2):e0263417. doi: 10.1371/journal.pone.0263417 (PMC8880763; doi:10.1371/journal.pone.0263417)
Supplement: S1 Table — (DOCX) [file pone.0263417.s001.docx]

**Supplementary Table 1**. Independent predictors of inpatient mortality in patients with COVID-19 across in different study periods separately.

|  | Peak 1 |  | Peak 2 |  | Off peaks |  |
| --- | --- | --- | --- | --- | --- | --- |
| predictor | OR (95% CI) | p | OR (95% CI) | p | OR (95% CI) | p |
| Age, per 5 years | 1.23  (1.16 - 1.31) | <.0001 | 1.15  (1.09 - 1.22) | <.0001 | 1.24  (1.15 - 1.34) | <.0001 |
| Male gender (ref: female) | 1.43  (1.05 - 1.95) | 0.0247 | 1.18  (0.88 - 1.57) | 0.27 | 1.78  (1.17 - 2.7) | 0.0066 |
| Hispanic (ref: white) | 0.64  (0.42 - 0.97) | 0.0343 | 1.24  (0.85 - 1.81) | 0.28 | 1.78  (1.07 - 2.96) | 0.0265 |
| Black (ref: white) | 0.84  (0.54 - 1.31) | 0.44 | 0.71  (0.45 - 1.13) | 0.15 | 1.25  (0.72 - 2.19) | 0.43 |
| Asian (ref: white) | 1.02  (0.62 - 1.67) | 0.95 | 0.82  (0.53 - 1.28) | 0.38 | 1.37  (0.7 - 2.68) | 0.35 |
| Morbid obesity * | 2.79  (1.61 - 4.81) | 0.0002 | 1.21  (0.66 - 2.23) | 0.54 | 3.45  (1.73 - 6.89) | 0.0004 |
| ECI 6-10 (ref: ECI ≤ 5) | 2.75  (1.44 - 5.26) | 0.0023 | 4.74  (2.4 - 9.33) | <.0001 | 2.26  (0.90 - 5.66) | 0.08 |
| ECI 11-17 (ref: ECI ≤ 5) | 5.81  (3.29 - 10.25) | <.0001 | 9.04  (4.91 - 16.64) | <.0001 | 5.99  (2.83 - 12.67) | <.0001 |
| ECI 18-27 (ref: ECI ≤ 5) | 7.32  (4.12 - 12.99) | <.0001 | 14.05  (7.61 - 25.96) | <.0001 | 10.51  (4.97 - 22.24) | <.0001 |
| ECI ≥ 28 (ref: ECI ≤ 5) | 12.87  (7.2 - 23) | <.0001 | 20.03  (10.57 - 37.97) | <.0001 | 21.88  (10.26 - 46.68) | <.0001 |
| Oxygen saturation at admission ≤ 90% | 2.32  (1.67 - 3.22) | <.0001 | 2.78  (2.05 - 3.76) | <.0001 | 3.50  (2.29 - 5.35) | <.0001 |
| High risk (qSOFA>2 at admission) | 2.29  (1.51 - 3.48) | 0.0001 | 1.80  (1.22 - 2.67) | 0.0033 | 2.03  (1.17 - 3.52) | 0.0122 |
