# Supplementary material for: Outcomes of hospitalized patients with COVID-19 during the course of the pandemic in a fully integrated health system
Source: PLoS One. 2022 Feb 25;17(2):e0263417. doi: 10.1371/journal.pone.0263417 (PMC8880763; doi:10.1371/journal.pone.0263417)
Supplement: S2 Table — (DOCX) [file pone.0263417.s002.docx]

**Supplementary Table 2.** Comparison of clinico-demographic parameters and outcomes in patients of different races/ethnicities hospitalized with COVID-19.

|  | White | Black | Hispanic | Asian | Other |
| --- | --- | --- | --- | --- | --- |
| N | 1763 | 1048 | 2771 | 711 | 454 |
| Period of admission: |  |  |  |  |  |
| Peak 1 | 437 (24.8%) | 298 (28.4%) | 1054 (38.0%) | 181 (25.5%) | 87 (19.2%) |
| Plateau | 269 (15.3%) | 215 (20.5%) | 721 (26.0%) | 90 (12.7%) | 97 (21.4%) |
| Peak 2 | 739 (41.9%) | 323 (30.8%) | 703 (25.4%) | 311 (43.7%) | 187 (41.2%) |
| Decline | 318 (18.0%) | 212 (20.2%) | 293 (10.6%) | 129 (18.1%) | 83 (18.3%) |
| Age, years | 68.9 ± 18.3 | 58.8 ± 17.4 | 49.3 ± 16.9 | 63.2 ± 16.7 | 57.3 ± 17.1 |
| Male | 942 (53.4%) | 502 (47.9%) | 1375 (49.6%) | 394 (55.4%) | 249 (54.8%) |
| Congregated living | 379 (22.4%) | 91 (9.1%) | 38 (1.4%) | 34 (4.9%) | 17 (3.8%) |
| BMI, kg/m2 | 28.9 ± 7.3 | 31.7 ± 8.8 | 31.1 ± 7.0 | 25.9 ± 5.3 | 29.6 ± 6.5 |
| Charlson’s comorbidity index (CCI) | 5.54 ± 3.76 | 4.65 ± 3.87 | 2.25 ± 2.63 | 4.18 ± 3.23 | 3.68 ± 3.37 |
| CCI = 0 | 157 (8.9%) | 136 (13.0%) | 894 (32.3%) | 84 (11.8%) | 84 (18.5%) |
| CCI = 1 | 142 (8.1%) | 140 (13.4%) | 529 (19.1%) | 77 (10.8%) | 58 (12.8%) |
| CCI = 2 | 156 (8.8%) | 117 (11.2%) | 407 (14.7%) | 100 (14.1%) | 62 (13.7%) |
| CCI = 3 or 4 | 300 (17.0%) | 193 (18.4%) | 486 (17.5%) | 169 (23.8%) | 101 (22.2%) |
| CCI = 5-8 | 605 (34.3%) | 275 (26.2%) | 353 (12.7%) | 199 (28.0%) | 106 (23.3%) |
| CCI >= 9 | 403 (22.9%) | 187 (17.8%) | 102 (3.7%) | 82 (11.5%) | 43 (9.5%) |
| Elixhauser comorbidity index (ECI) | 14.7 ± 12.1 | 12.4 ± 12.4 | 6.60 ± 9.16 | 11.9 ± 11.4 | 10.2 ± 11.3 |
| ECI <= 0 | 232 (13.2%) | 190 (18.1%) | 984 (35.5%) | 116 (16.3%) | 111 (24.4%) |
| 1 <= ECI <= 5 | 269 (15.3%) | 231 (22.0%) | 739 (26.7%) | 164 (23.1%) | 97 (21.4%) |
| 6 <= ECI <= 10 | 236 (13.4%) | 136 (13.0%) | 338 (12.2%) | 119 (16.7%) | 67 (14.8%) |
| 11 <= ECI <= 17 | 362 (20.5%) | 161 (15.4%) | 381 (13.7%) | 124 (17.4%) | 78 (17.2%) |
| 18 <= ECI <= 27 | 380 (21.6%) | 190 (18.1%) | 210 (7.6%) | 113 (15.9%) | 63 (13.9%) |
| ECI >= 28 | 284 (16.1%) | 140 (13.4%) | 119 (4.3%) | 75 (10.5%) | 38 (8.4%) |
| **Resource utilization and outcomes** |  |  |  |  |  |
| Length of stay, days | 9.04 ± 9.58 | 9.59 ± 9.95 | 8.66 ± 10.61 | 10.3 ± 10.0 | 9.40 ± 10.44 |
| Admitted to ICU | 463 (26.3%) | 336 (32.1%) | 787 (28.4%) | 222 (31.2%) | 130 (28.6%) |
| Received mechanical ventilation | 179 (10.2%) | 128 (12.2%) | 304 (11.0%) | 111 (15.6%) | 60 (13.2%) |
| Received ECMO | 10 (0.6%) | 6 (0.6%) | 31 (1.1%) | 5 (0.7%) | 3 (0.7%) |
| Discharged to: |  |  |  |  |  |
| Short-term care facility | 13 (0.7%) | 10 (1.0%) | 23 (0.8%) | 7 (1.0%) | 7 (1.5%) |
| Long-term care facility | 361 (20.5%) | 132 (12.6%) | 89 (3.2%) | 66 (9.3%) | 33 (7.3%) |
| Home | 997 (56.6%) | 767 (73.2%) | 2465 (89.0%) | 527 (74.1%) | 355 (78.2%) |
| Hospice care | 84 (4.8%) | 24 (2.3%) | 17 (0.6%) | 18 (2.5%) | 5 (1.1%) |
| Died | 308 (17.5%) | 115 (11.0%) | 177 (6.4%) | 93 (13.1%) | 54 (11.9%) |
