# Supplementary material for: Outcomes of hospitalized patients with COVID-19 during the course of the pandemic in a fully integrated health system
Source: PLoS One. 2022 Feb 25;17(2):e0263417. doi: 10.1371/journal.pone.0263417 (PMC8880763; doi:10.1371/journal.pone.0263417)
Supplement: S3 Table — (DOCX) [file pone.0263417.s003.docx]

**Supplementary Table 3.** Comparison of clinico-demographic parameters and outcomes in Hispanic vs. non-Hispanic patients hospitalized with COVID-19.

|  | Hispanic | non-Hispanic | Prob |
| --- | --- | --- | --- |
| N | 2771 | 3900 |  |
| **Period of admission:** |  |  |  |
| Peak 1 | 1054 (38.0%) | 983 (25.2%) | <.0001 |
| Plateau | 721 (26.0%) | 661 (16.9%) | <.0001 |
| Peak 2 | 703 (25.4%) | 1507 (38.6%) | <.0001 |
| Decline | 293 (10.6%) | 749 (19.2%) | <.0001 |
| **Demographics and clinical:** |  |  |  |
| Age, years (mean ± SD) | 49.3 ± 16.9 | 63.9 ± 18.3 | <.0001 |
| Age < 45 | 1141 (41.2%) | 624 (16.0%) | <.0001 |
| Age 45-54 | 597 (21.5%) | 540 (13.8%) | <.0001 |
| Age 55-64 | 492 (17.8%) | 718 (18.4%) | 0.4939 |
| Age 65-74 | 316 (11.4%) | 772 (19.8%) | <.0001 |
| Age 75-84 | 164 (5.9%) | 732 (18.8%) | <.0001 |
| Age 85-94 | 56 (2.0%) | 431 (11.1%) | <.0001 |
| Age >= 95 | 5 (0.2%) | 83 (2.1%) | <.0001 |
| Male | 1375 (49.6%) | 2052 (52.6%) | 0.0159 |
| Congregated living | 38 (1.4%) | 514 (13.7%) | <.0001 |
| BMI, kg/m2 | 31.1 ± 7.0 | 29.2 ± 7.6 | <.0001 |
| BMI < 18 | 12 (0.5%) | 106 (2.9%) | <.0001 |
| Obesity (BMI >= 30) | 1316 (49.9%) | 1428 (38.6%) | <.0001 |
| Morbid obesity (BMI >= 40) | 242 (9.2%) | 307 (8.3%) | 0.2197 |
| Charlson’s comorbidity index (CCI) | 2.25 ± 2.63 | 4.85 ± 3.72 | <.0001 |
| CCI = 0 | 894 (32.3%) | 454 (11.6%) | <.0001 |
| CCI = 1 | 529 (19.1%) | 408 (10.5%) | <.0001 |
| CCI = 2 | 407 (14.7%) | 425 (10.9%) | <.0001 |
| CCI = 3 or 4 | 486 (17.5%) | 749 (19.2%) | 0.0842 |
| CCI = 5-8 | 353 (12.7%) | 1165 (29.9%) | <.0001 |
| CCI >= 9 | 102 (3.7%) | 699 (17.9%) | <.0001 |
| Elixhauser comorbidity index (ECI) | 6.60 ± 9.16 | 13.1 ± 12.0 | <.0001 |
| ECI <= 0 | 984 (35.5%) | 629 (16.1%) | <.0001 |
| 1 <= ECI <= 5 | 739 (26.7%) | 750 (19.2%) | <.0001 |
| 6 <= ECI <= 10 | 338 (12.2%) | 547 (14.0%) | 0.0301 |
| 11 <= ECI <= 17 | 381 (13.7%) | 713 (18.3%) | <.0001 |
| 18 <= ECI <= 27 | 210 (7.6%) | 734 (18.8%) | <.0001 |
| ECI >= 28 | 119 (4.3%) | 527 (13.5%) | <.0001 |
| **Vitals at admission:** |  |  |  |
| Blood pressure diastolic, mmHg | 72.9 ± 12.4 | 72.3 ± 13.2 | 0.1132 |
| Blood pressure systolic, mmHg | 126.6 ± 21.5 | 130.3 ± 24.2 | <.0001 |
| Temperature, degrees F | 99.0 ± 1.5 | 98.8 ± 1.5 | <.0001 |
| Heart rate per minute | 90.2 ± 19.5 | 87.7 ± 19.7 | <.0001 |
| Respiratory rate per minute | 22.5 ± 7.3 | 22.3 ± 7.9 | 0.5408 |
| Oxygen saturation, % | 92.6 ± 7.5 | 93.2 ± 7.0 | 0.0009 |
| Oxygen saturation <= 90% | 669 (24.5%) | 840 (21.6%) | 0.0060 |
| On supplemental oxygen | 2414 (88.3%) | 3183 (81.8%) | <.0001 |
| High risk (qSOFA = 2 or 3) | 128 (4.7%) | 349 (9.4%) | <.0001 |
| **Laboratory parameters** |  |  |  |
| ALT, U/L | 56.9 ± 77.1 | 44.0 ± 72.4 | <.0001 |
| AST, U/L | 58.3 ± 73.1 | 56.5 ± 94.9 | <.0001 |
| Bicarbonate, mEq | 22.3 ± 3.7 | 22.9 ± 4.2 | <.0001 |
| Serum creatinine, mg/dL | 1.24 ± 1.76 | 1.55 ± 1.77 | <.0001 |
| C-reactive protein, mg/L | 12.7 ± 9.1 | 10.5 ± 8.3 | <.0001 |
| D-dimer, mg/L | 1.76 ± 5.45 | 2.48 ± 3.78 | <.0001 |
| Ferritin, ng/mL | 1066.9 ± 1430.0 | 1239.6 ± 2006.6 | 0.5578 |
| Hemoglobin, g/dL | 13.2 ± 2.0 | 12.9 ± 2.4 | <.0001 |
| Absolute lymphocyte count | 1.63 ± 6.61 | 1.69 ± 10.28 | <.0001 |
| Platelet, 109/L | 243.7 ± 91.3 | 222.5 ± 95.3 | <.0001 |
| Total Bilirubin, mg/dL | 0.658 ± 0.897 | 0.697 ± 0.981 | <.0001 |
| White blood count, 109/L | 8.87 ± 4.72 | 8.71 ± 20.86 | <.0001 |
| **Resource utilization:** |  |  |  |
| Length of stay, days | 8.66 ± 10.61 | 9.47 ± 9.86 | <.0001 |
| Admitted to ICU | 787 (28.4%) | 1138 (29.2%) | 0.4894 |
| Received mechanical ventilation | 304 (11.0%) | 473 (12.1%) | 0.1464 |
| Received ECMO | 31 (1.1%) | 23 (0.6%) | 0.0175 |
| Has hospice status at some point | 21 (0.8%) | 219 (5.6%) | <.0001 |
| **Discharged to:** |  |  |  |
| Short-term care facility | 23 (0.8%) | 35 (0.9%) | 0.7701 |
| Long-term care facility | 89 (3.2%) | 587 (15.1%) | <.0001 |
| Home | 2465 (89.0%) | 2591 (66.4%) | <.0001 |
| Hospice care | 17 (0.6%) | 129 (3.3%) | <.0001 |
| Died | 177 (6.4%) | 558 (14.3%) | <.0001 |
