# Supplementary material for: Outcomes of hospitalized patients with COVID-19 during the course of the pandemic in a fully integrated health system
Source: PLoS One. 2022 Feb 25;17(2):e0263417. doi: 10.1371/journal.pone.0263417 (PMC8880763; doi:10.1371/journal.pone.0263417)
Supplement: S4 Table — (DOCX) [file pone.0263417.s004.docx]

**Supplementary Table 4.** Comparison of outcomes of patients with different baseline comorbidity scores.

| By Charlson’s comorbidity index (CCI) | CCI 0-1 | CCI 2-4 | CCI 5-8 | CCI 9+ |
| --- | --- | --- | --- | --- |
| N | 2344 | 2132 | 1566 | 823 |
| Age, years | 39.0 ± 10.8 | 59.5 ± 11.9 | 73.2 ± 13.6 | 77.9 ± 11.0 |
| Male | 1109 (47.3%) | 1205 (56.5%) | 810 (51.7%) | 398 (48.4%) |
| White | 299 (13.4%) | 456 (22.1%) | 605 (39.4%) | 403 (49.4%) |
| Black | 276 (12.3%) | 310 (15.0%) | 275 (17.9%) | 187 (22.9%) |
| Hispanic | 1423 (62.3%) | 893 (43.2%) | 353 (23.3%) | 102 (12.7%) |
| Asian | 161 (7.2%) | 269 (13.0%) | 199 (13.0%) | 82 (10.0%) |
| Other race/ethnicity | 142 (6.3%) | 163 (7.9%) | 106 (6.9%) | 43 (5.3%) |
| Congregated living | 15 (0.7%) | 65 (3.1%) | 278 (18.1%) | 214 (26.8%) |
| Length of stay, days | 5.74 ± 6.51 | 10.4 ± 11.8 | 11.2 ± 10.8 | 11.2 ± 10.3 |
| Admitted to ICU | 418 (17.8%) | 696 (32.6%) | 568 (36.3%) | 295 (35.8%) |
| Received mechanical ventilation | 108 (4.6%) | 308 (14.4%) | 260 (16.6%) | 115 (14.0%) |
| Received ECMO | 21 (0.9%) | 28 (1.3%) | 3 (0.2%) | 4 (0.5%) |
| Discharged to: |  |  |  |  |
| Short-term care facility | 19 (0.8%) | 25 (1.2%) | 12 (0.8%) | 4 (0.5%) |
| Long-term care facility | 21 (0.9%) | 140 (6.6%) | 324 (20.7%) | 208 (25.3%) |
| Home | 2274 (97.0%) | 1787 (83.8%) | 827 (52.8%) | 315 (38.3%) |
| Hospice care | 2 (0.1%) | 9 (0.4%) | 75 (4.8%) | 64 (7.8%) |
| Died | 28 (1.2%) | 171 (8.0%) | 328 (20.9%) | 232 (28.2%) |
| By Elixhauser’s comorbidity index (ECI) | ECI <= 5 | ECI 6-10 | ECI 11-27 | ECI >= 28 |
| N | 3199 | 911 | 2098 | 657 |
| Age, years | 48.3 ± 16.7 | 59.2 ± 16.9 | 67.2 ± 17.0 | 72.6 ± 14.5 |
| Male | 1509 (47.2%) | 513 (56.3%) | 1151 (54.9%) | 349 (53.1%) |
| White | 501 (16.4%) | 236 (26.6%) | 742 (36.2%) | 284 (43.4%) |
| Black | 421 (13.7%) | 136 (15.3%) | 351 (17.1%) | 140 (21.4%) |
| Hispanic | 1723 (55.5%) | 338 (38.2%) | 591 (29.0%) | 119 (18.4%) |
| Asian | 280 (9.1%) | 119 (13.4%) | 237 (11.6%) | 75 (11.5%) |
| Other race/ethnicity | 208 (6.8%) | 67 (7.6%) | 141 (6.9%) | 38 (5.8%) |
| Congregated living | 52 (1.7%) | 66 (7.4%) | 300 (14.7%) | 154 (24.2%) |
| Length of stay, days | 6.11 ± 6.37 | 9.23 ± 8.39 | 12.1 ± 12.3 | 13.8 ± 14.2 |
| Admitted to ICU | 578 (18.1%) | 278 (30.5%) | 816 (38.9%) | 305 (46.4%) |
| Received mechanical ventilation | 101 (3.2%) | 91 (10.0%) | 432 (20.6%) | 167 (25.4%) |
| Received ECMO | 13 (0.4%) | 5 (0.5%) | 23 (1.1%) | 15 (2.3%) |
| Discharged to: |  |  |  |  |
| Short-term care facility | 27 (0.8%) | 3 (0.3%) | 26 (1.2%) | 4 (0.6%) |
| Long-term care facility | 79 (2.5%) | 89 (9.8%) | 366 (17.4%) | 159 (24.2%) |
| Home | 3032 (94.8%) | 737 (80.9%) | 1203 (57.3%) | 231 (35.2%) |
| Hospice care | 12 (0.4%) | 12 (1.3%) | 89 (4.2%) | 37 (5.6%) |
| Died | 49 (1.5%) | 70 (7.7%) | 414 (19.7%) | 226 (34.4%) |
